# Pressure tuning of charge ordering in iron oxide

**DOI:** 10.1038/s41467-018-06457-x

**Published:** 2018-10-08

**Authors:** Sergey V. Ovsyannikov, Maxim Bykov, Elena Bykova, Konstantin Glazyrin, Rudra Sekhar Manna, Alexander A. Tsirlin, Valerio Cerantola, Ilya Kupenko, Alexander V. Kurnosov, Innokenty Kantor, Anna S. Pakhomova, Irina Chuvashova, Aleksandr I. Chumakov, Rudolf Rüffer, Catherine McCammon, Leonid S. Dubrovinsky

**Affiliations:** 1 Bayerisches Geoinstitut, Universität Bayreuth, Universitätsstrasse 30, D-95447 Bayreuth, Germany; 2 The Institute for Solid State Chemistry, Russian Academy of Sciences, Urals Division, 91 Pervomayskaya Str., Yekaterinburg, 620990 Russia; 3 Deutsches Elektronen-Synchrotron (DESY), D-22603 Hamburg, Germany; 4 Department of Physics, IIT Tirupati, Tirupati, 517506 India; 5 Experimental Physics VI, Center for Electronic Correlations and Magnetism, Institute of Physics, University of Augsburg, 86135 Augsburg, Germany; 6 ESRF—The European Synchrotron, CS40220, 38043 Grenoble Cedex 9, France; 7 Institut für Mineralogie, Universität Münster, Corrensstraße 24, D-48149 Münster, Germany; 8 Department of Physics, Technical University of Denmark, 2800 Kgs. Lyngby, Denmark

## Abstract

A Verwey-type charge-ordering transition in magnetite at 120 K leads to the formation of linear units of three iron ions with one shared electron, called trimerons. The recently-discovered iron pentoxide (Fe_4_O_5_) comprising mixed-valent iron cations at octahedral chains, demonstrates another unusual charge-ordering transition at 150 K involving competing formation of iron trimerons and dimerons. Here, we experimentally show that applied pressure can tune the charge-ordering pattern in Fe_4_O_5_ and strongly affect the ordering temperature. We report two charge-ordered phases, the first of which may comprise both dimeron and trimeron units, whereas, the second exhibits an overall dimerization involving both the octahedral and trigonal-prismatic chains of iron in the crystal structure. We link the dramatic change in the charge-ordering pattern in the second phase to redistribution of electrons between the octahedral and prismatic iron chains, and propose that the average oxidation state of the iron cations can pre-determine a charge-ordering pattern.

## Abstract (web summary)

The charge order transition of commonly known magnetite has only recently been unraveled. Here, the measurement of the low-temperature high-pressure phase diagram of a related material (Fe_4_O_5_) elucidates the interplay of average oxidation state and charge-ordering phenomena in the iron oxide family.

## Introduction

Iron oxides, composed of two of the most abundant elements in the Earth’s interior, are fundamentally important materials both for basic science and applied technologies^1^. The first-known magnetic mineral, magnetite (Fe_3_O_4_), was the only simple mixed-valent iron oxide known until recently, and for this reason it is a key model system for investigation of Fe^2+^/Fe^3+^ interplay and related physical phenomena. It was discovered that at 120 K magnetite exhibits an abrupt transition of a metal-insulator-type (the so-called Verwey transition), which is supposed to result from enigmatic charge ordering at the octahedral sites of its cubic spinel structure^2^. Numerous studies were aimed to comprehend the nature and mechanisms of this transition^3^. Only recently, the elusive charge-ordering pattern in the Verwey phase of magnetite below 120 K was finally solved by means of single crystal X-ray diffraction, and a novel type of quasi-particle consisting of linear units of three iron ions with one shared electron, called a trimeron, was proposed^4,5^. This charge ordering is highly unusual and implies a more intricate underlying phenomenon^6–8^ than ordering of separate Fe^2+^ and Fe^3+^ ions that takes place, for example, in TbBaFe_2_O_5_^9,10^ or charge ordering during the course of charge disproportionation^11^.

It was commonly thought that all of the simplest iron oxides fall within three basic stoichiometries, such as FeO, Fe_3_O_4_, and Fe_2_O_3_. A recent series of high-pressure high-temperature (HP-HT) studies using single crystal X-ray diffraction methods discovered and identified a number of novel iron oxides, e.g., Fe_4_O_5_^12,13^, Fe_5_O_6_^14,15^, Fe_13_O_19_^16^, Fe_5_O_7_^17^, Fe_25_O_32_^17^, FeO_2_^18–21^, Fe_7_O_9_^22^, and a new monoclinic polymorph of Fe_2_O_3_ which can be stable at ambient conditions^23^. Some of the novel oxides, like Fe_4_O_5_, Fe_5_O_6_, and Fe_7_O_9_, were also found to be readily recoverable at ambient conditions. These discoveries open a portal to mixed-valent iron oxides and motivate investigation of their physical properties and potential for emergent innovative applications. Fe_4_O_5_ can be synthesized at moderate HP-HT conditions of about 10 GPa and 1000 °C and has appeared to be the most common of the above high-pressure iron oxides. It crystallizes in an orthorhombic CaFe_3_O_5_-type structure, one of the known Ca-ferrite (${\mathrm{CaFe}}_n^{2 + }{\mathrm{Fe}}_2^{3 + }{\mathrm{O}}_{4 + n}$, *n* = 1, 2, 3, etc.) phases^24,25^, and this fact could explain the phase and chemical stability of Fe_4_O_5_ and its derivatives, (M,Fe)_2_Fe_2_O_5_ (M—Mg, Cr, Mn)^26–31^ at ambient conditions. The crystal structure of Fe_4_O_5_ comprises chains of both trigonal prisms Fe1 (this crystallographic position is denoted here as Fe1) with shared triangular faces, occupied by Fe^2+^ cations, and an octahedral network occupied by mixed Fe^2+^/Fe^3+^ cations and consisting of (i) single chains of octahedra sharing opposite edges (this crystallographic position is denoted here as Fe2), and (ii) double chains of octahedra composed of two chains similar to the above Fe2 ones, but attached together side-by-side via two other octahedron edges (this crystallographic position is denoted here as Fe3)^12^. Like magnetite, Fe_4_O_5_ shows a charge-ordering transition below 150 K involving the competing formation of iron trimers and dimers (two Fe ions with one shared electron) at the octahedral network^32^. It is interesting to note that a recent study identified coexisting trigonal and dimer-like charge ordering patterns in layered α-RuCl_3_^33^.

In this work, we synthesize high-quality single crystals of Fe_4_O_5_ and investigate the effect of high pressure on charge ordering in Fe_4_O_5_ at low temperature using single crystal X-ray diffraction, Mössbauer spectroscopy, and magnetization measurements. We find that cold compression of Fe_4_O_5_ stimulates electron transfer between iron cations of different coordination and leads to the formation of novel charge-ordered phases.

## Results

### Phase III and two options for its crystal structure

**Fig. 1:**
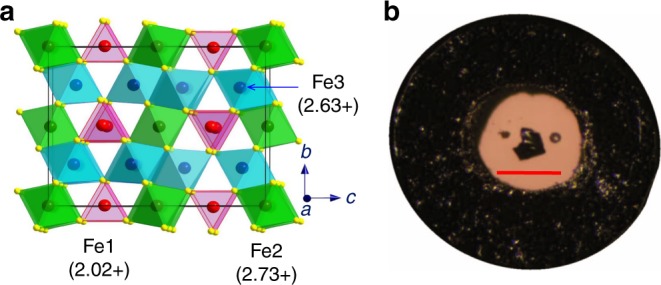
Crystal structure and image of the Fe_4_O_5_ crystal. **a** Orthorhombic crystal structure of Fe_4_O_5_ at ambient conditions showing the different crystallographic positions for iron atoms (Fe1, Fe2, and Fe3) in different colors. The bond valence sum values are indicated near the cations. **b** Image of a single crystal of Fe_4_O_5_ taken through one diamond window. A rhenium gasket of about 40 µm thickness with a circular hole of about 150 µm diameter drilled in its central part is bridged between the two opposing diamond anvils. The light background color of the central hole is due to light passing through the underlying diamond anvil. The central hole (working area) accommodates the Fe_4_O_5_ single crystal (in the center) and two small pressure markers, Au and ruby, located on the left and right sides of the crystal, respectively. The central hole is filled with a transparent neon pressure-transmitting medium. The scale bar inside the central hole corresponds to 100 µm

**Fig. 2:**
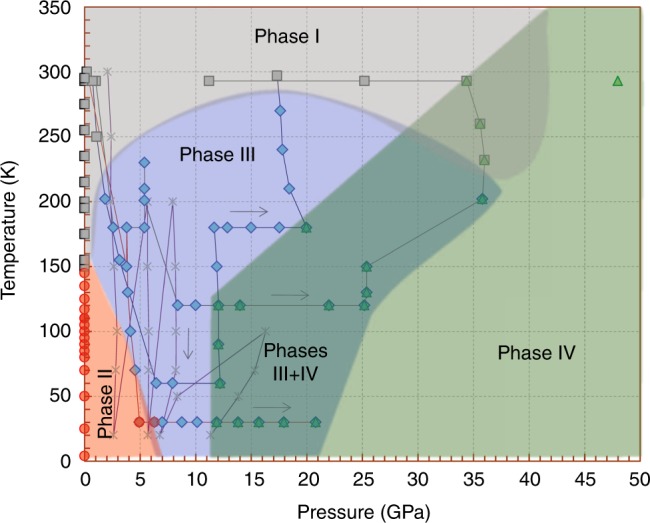
High-pressure low-temperature phase diagram of Fe_4_O_5_. The diagram is based on the single crystal X-ray diffraction experiments. Experimental points on the diagram corresponding to different phases are shown in different symbols. The shaded areas show the proposed stability regions of the phases. The lines and arrows show the directions of the pressure and temperature variation during the experiments. Since the basic structural reflections of Fe_4_O_5_-I and Fe_4_O_5_-III structures are identical (Fig. 3), the region of their co-existence could not be properly delineated. One cannot rule out that the region of Fe_4_O_5_-III in the phase diagram might be a superposition of stability ranges of Fe_4_O_5_-III-a and Fe_4_O_5_-III-b phases

**Fig. 3:**
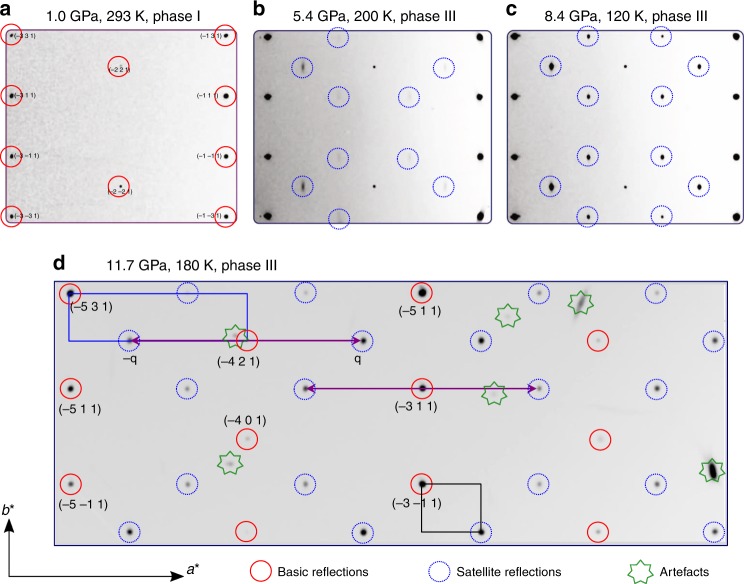
Examples of reciprocal lattice planes (*hkl*) of Fe_4_O_5_. These plots correspond to different pressure and temperature conditions (indicated for each plot). **a** Basic reflections corresponding to the original Fe_4_O_5_-I structure, highlighted by red circles. **b**, **c** Emergence of superlattice reflections, highlighted by blue circles (in **b** these reflections are rather weak and blurred, but in **c** they are strong and clear). **d** Reconstruction of (*hkl*) reciprocal lattice planes, which reveals two possible unit cells of Fe_4_O_5_-III. The first option is an orthorhombic *C*-centered “average” cell, shown in the upper left corner together with its modulation wave vector **q** (Fe_4_O_5_-III-b). The second option is a *C*-centered unit cell with a tripled lattice parameter *a*, compared to the average cell (Fe_4_O_5_-III-a), highlighted at the bottom of the plot

**Fig. 4:**
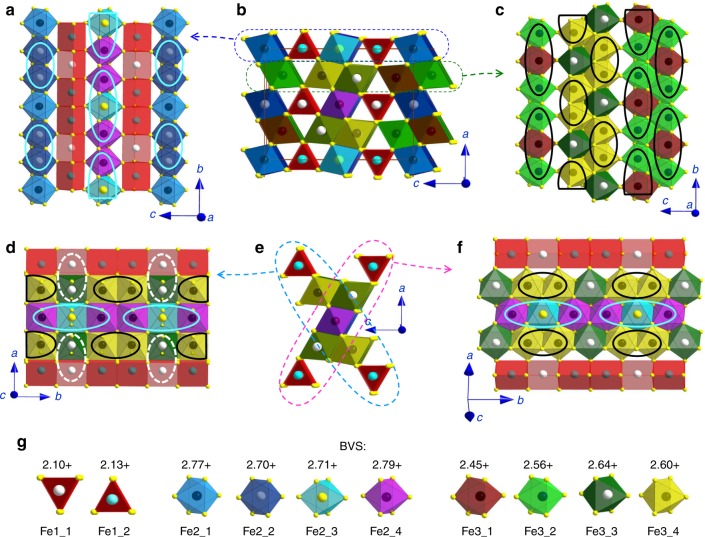
Monoclinic crystal structure of Fe_4_O_5_-III-a. This structure has *C*2*/m* symmetry and plots are based on the crystal structure data refined at 11.7 GPa and 180 K. **a** Projection of the trigonal prisms Fe1 and single chains of Fe2 octahedra. **b** The unit cell projected down the *b*-axis. Thin solid lines indicate the unit cell edges. **c** Projection of the double chains of Fe3 octahedra. **d** Projection down the *c*-axis of one of the two diagonal ribbons shown in **e**. **e** Two characteristic diagonal ribbons consisting of five iron chains each. **f** Projection of one of two diagonal ribbons shown in **e**. Solid ellipsoids are dimers and trimers formed in the chains of octahedrally-coordinated iron. Dotted ellipsoids highlight Fe3–Fe1 pairs with shortened Fe–Fe length. Different crystallographic positions are shown in different colors. **g** Bond valence sums (BVS) values of all the iron cations in the crystal structure

**Fig. 5:**
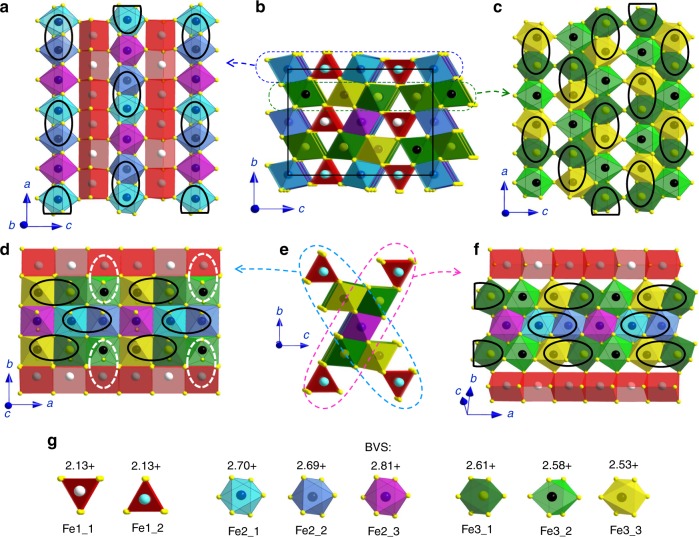
Orthorhombic crystal structure of Fe_4_O_5_-III-b. The structure has *C*222_1_ symmetry and plots are based on the crystal structure data refined at 11.7 GPa and 180 K. **a** Projection of the trigonal prisms Fe1 and single-chains of Fe2 octahedra. **b** The unit cell projected down the *a*-axis. Thin solid lines indicate the unit cell edges. **c** Projection of the double chains of Fe3 octahedra. **d** Projection down the *c*-axis of one of the two diagonal ribbons shown in **e**. **e** Two characteristic diagonal ribbons consisting of five iron chains each. **f** Projection of one of the two diagonal ribbons shown in **e**. Solid ellipsoids are dimers formed in the chains of octahedrally-coordinated iron. Dotted ellipsoids highlight Fe3–Fe1 pairs with shortened Fe–Fe length. Different crystallographic positions are shown in different colors. **g** Bond valence sums (BVS) values of all the iron cations in the crystal structure

In all experiments, we start from normal conditions (Fe_4_O_5_-I phase) (Figs. 1a and 2) and gradually decrease the temperature in the cryostat. At about 200 K and 2 GPa, we observe the appearance of weak and blurred superlattice reflections, indicating the emergence of scattered fragments of a charge-ordering pattern (Fig. 3b). With further temperature decrease and pressure increase, these reflections become progressively stronger and clearer (Fig. 3c). We verify that these reflections do not belong to the earlier-reported charge-ordered Fe_4_O_5_-II phase^32^, and label this phase as Fe_4_O_5_-III. Meanwhile, the presence of Fe_4_O_5_-II is noticeable at 5–7 GPa at the lowest temperature point of 30 K (Fig. 2). Upon heating at about 18 GPa, we can follow the superlattice reflections of Fe_4_O_5_-III up to at least 270 K (Fig. 2). Thus, pressure strongly enhances the temperature of the charge-ordering transition, compared to 150 K at ambient pressure^32^. We can index the single-crystal diffraction patterns of Fe_4_O_5_-III in either orthorhombic or monoclinic unit cells (Fig. 3d). Eventually, we determine two candidate crystal structures, namely, Fe_4_O_5_-III-a with a monoclinic *C*2*/m* lattice (Fig. 4) and Fe_4_O_5_-III-b with an orthorhombic *C*222_1_ unit cell (Fig. 5), which can equally well fit the experimental X-ray diffraction patterns (Table 1 and Supplementary Table 1). Compared to the original structure (Fe_4_O_5_-I), the crystallographic sites of the iron cations occupying the trigonal prisms in the crystal structures of both Fe_4_O_5_-III-a and Fe_4_O_5_-III-b are split into two slightly non-equivalent Fe1_1 and Fe1_2 positions; likewise the octahedral sites Fe2 and Fe3 are split into multiple non-equivalent sites, highlighted in different colors in Figs. 4 and 5. Note here that the unusual incommensurately-modulated character of the crystal structure of Fe_4_O_5_-II, reported in previous work^32^, could result from a lattice instability related to competition between the Fe_4_O_5_-III-a and Fe_4_O_5_-III-b phases. A similar type of incommensurability was observed, for example, in the spin-Peierls compound, TiPO_4_^34,35^.

**Table 1:** Unit cell parameters of different phases of Fe_4_O_5_

Details of crystal structures	Phases				
	Fe_4_O_5_-I	Fe_4_O_5_-III-a	Fe_4_O_5_-III-b	Fe_4_O_5_-IV (LT)	Fe_4_O_5_-IV (HT)^a^
Pressure (GPa)	Ambient	11.7	11.7	25.2	48
Temperature (K)	293	180	180	120	296
Crystal system	Orthorhombic	Monoclinic	Orthorhombic	Monoclinic	Monoclinic
Space group (No.)	*Cmcm* (No. 63)	*C*2/*m* (No. 12)	*C*222_1_ (No. 20)	*P*2_1_/*m* (No. 11)	*P*2_1_/*m* (No. 11)
Lattice parameter, *a* (Å)	2.89200(5)	9.675(4)	8.4492(3)	5.0145(10)	4.9408(10)
Lattice parameter, *b* (Å)	9.7979(2)	8.4493(3)	9.6750(4)	12.1155(18)	11.7880(18)
Lattice parameter, *c* (Å)	12.583(2)	12.328(7)	12.328(7)	5.4282(4)	5.3287(4)
*β* (°)		90.0(1)		105.582(11)	105.320(11)
Unit cell volume, *V* (Å^3^)	356.54 (7)	1007.8 (6)	1007.7 (6)	317.66(8)	299.33(8)
*Z*	4	12	12	4	4
Calculated density (g/cm^3^)	5.65138	5.99868	5.99875	6.34361	6.73215

^a^This phase appeared after laser heating

In magnetite, the formation of iron trimers in its charge-ordered phase is perfectly traced by anomalous shortening of some Fe–Fe distances as found by single crystal X-ray diffraction method^4^; hence we also apply this approach to Fe_4_O_5_. The octahedral chains occupied by mixed-valent iron cations show pronounced shortening of distances between some neighboring Fe atoms; whereas the prismatic chains of iron in both Fe_4_O_5_-III-a and Fe_4_O_5_-III-b structures are characterized by nearly equal Fe–Fe distances. For example, at 11.7 GPa and 180 K, the Fe–Fe distances in the prismatic chains of the Fe_4_O_5_-III-a structure show a periodicity consisting of one 2.8143 Å and two 2.8176 Å distances; since these two values are very similar, we cannot draw any conclusions about the formation of coupled units in these chains. Compared to the average Fe–Fe distance of ~2.8165 Å along the same crystallographic direction (*b*-axis), the distances between two neighboring octahedrally-coordinated Fe2_2 atoms in the single chains of Fe2 octahedra are reduced, to 2.6597 Å, and likewise those between Fe3_4 atoms in the double chains of Fe3 octahedra are reduced to 2.7279 Å (Supplementary Table 2). These dramatic shortenings in the distances suggest the formation of dimers (Fig. 4a, c). In the other octahedral chains of this Fe_4_O_5_-III-a phase, equal contractions in two neighboring Fe–Fe distances indicate the formation of trimers.

The Fe_4_O_5_-III-b structure exhibits similar features. Its prismatic iron chains show a periodicity in the Fe–Fe bond lengths consisting of one 2.8118 Å and two 2.8189 Å distances, where the difference between the two is too small to allow any conclusions about the formation of any bonded units (Fig. 5). In contrast to Fe_4_O_5_-III-a, the Fe_4_O_5_-III-b structure does not contain any trimers (Fig. 5). Both octahedral chains of the Fe_4_O_5_-III-b structure exhibit only dimers with a Fe–Fe bond length of 2.6777 Å in the single chains of Fe2 octahedra, and 2.7387 Å in the double-chains of Fe3 octahedra (Supplementary Table 2). Both Fe_4_O_5_-III-a and Fe_4_O_5_-III-b crystal structures show a strong rapprochement between free Fe3 atoms (Fe3_3 in phase III-a and Fe3_2 in phase III-b, which are unincorporated into the dimer and trimer units) and the neighboring Fe1 atoms in the prisms, which are connected to these Fe3 octahedra via one shared edge (these Fe3–Fe1 pairs are highlighted as white dashed ellipsoids in Figs. 4d and 5d). The Fe–Fe distances in these Fe3–Fe1 pairs become nearly the same as the average Fe–Fe distances in both prismatic and octahedral chains (Supplementary Table 2), thereby suggesting an enhancement of interactions between the iron ions occupying the prisms and octahedra.

We analyze the Fe–O bond lengths of all iron cations in both Fe_4_O_5_-III structures using a common bond valence sum (BVS) method (see Experimental Details)^36^. We estimate the average BVS values of the Fe1 atoms sitting in the prisms as +2.11 and +2.13 in Fe_4_O_5_-III-a and Fe_4_O_5_-III-b structures, respectively. The BVS analysis suggests that the dimers formed at the double chains of Fe3 octahedra of both structures consist of moderately overcharged valence-mixed Fe^2.5+^ ions; whereas, the dimers formed at the single-chain Fe2 octahedra have a charge as high as +5.4 (Figs. 4 and 5). Likewise, this analysis shows that, compared to a combination of one Fe^2+^ and two Fe^3+^ ions, the trimers in the Fe_4_O_5_-III-a structure are either strongly overcharged (+8.29 for the single chains of Fe2 octahedra) or strongly undercharged (+7.57 for the double chains of Fe3 octahedra) (Fig. 4).

### Phase IV and charge ordering in chains of trigonal prisms

In all three experimental runs at pressures above 12 GPa and temperatures below 150 K, we observe the appearance of additional structural reflections (Supplementary Fig. 1), suggesting the beginning of another transition to a further phase, labeled as Fe_4_O_5_-IV (Fig. 2). Below 150 K this transition is nearly complete at 25 GPa, but at slightly higher temperature (200 K), minor traces of Fe_4_O_5_-III are still observable even at 36 GPa (Fig. 2). Upon heating to room temperature at 36 GPa, Fe_4_O_5_-IV gradually transforms to the original Fe_4_O_5_-I phase; meanwhile, a noticeable fraction of Fe_4_O_5_-IV persists even at 293 K (Fig. 2).

**Fig. 6:**
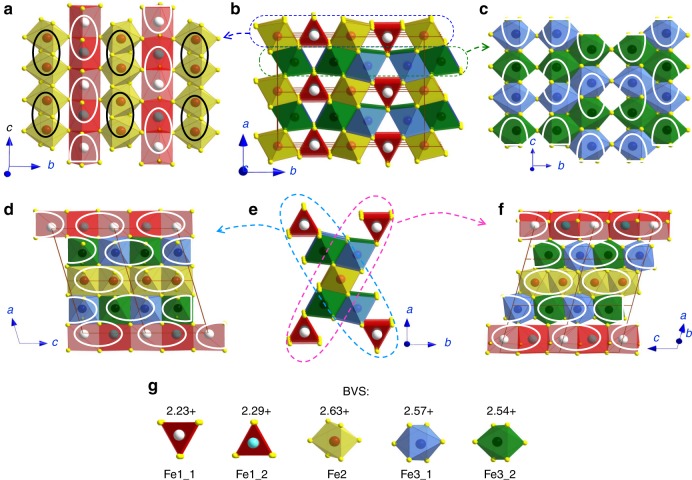
Monoclinic crystal structure of Fe_4_O_5_-IV. The structure has *P*2_1_/*m* symmetry and plots are based on the crystal structure data refined at 25.2 GPa and at 120 K. **a** Projection of the trigonal prisms Fe1 and single chains of Fe2 octahedra. **b** Two unit cells projected down the *c*-axis. Thin solid lines indicate the unit cell edges. **c** Projection of the double chains of Fe3 octahedra. **d** Projection down the *b*-axis of one of the two diagonal ribbons shown in **e**. **e** Two characteristic diagonal ribbons consisting of five iron chains each. **f** Projection down the *b*-axis of one of the two diagonal ribbons shown in **e**. Solid ellipsoids are dimers formed in all iron chains. Different crystallographic positions are shown in different colors. **g** Bond valence sums (BVS) values of all the iron cations in the crystal structure

The single crystal X-ray diffraction images of Fe_4_O_5_ collected at 25.2 GPa and 120 K correspond to almost pure Fe_4_O_5_-IV (Supplementary Fig. 1), and we determine its monoclinic *P*2_1_/*m* symmetry and atomic positions. The structure shows an overall dimerization along the *c*-axis involving both octahedral and prismatic chains (Fig. 6). We calculate BVS values of the iron ions in this structure using the above method^36^, and find an average value for the prismatic Fe1 atoms of +2.26. This value deviates from +2 established for these prismatic sites in the original Fe_4_O_5_-I phase^12,32^, and taking into account the emergence of dimeric ordering in these chains, we conclude that the prismatic sites in Fe_4_O_5_-IV are filled with mixed-valent iron cations. In the same way, the average BVS values of the iron in the single chains of Fe2 and double chains of Fe3 octahedra in Fe_4_O_5_-IV are found as +2.63 and +2.554, respectively (Fig. 6). These values are also consistent with the dimeric ordering in the chains. These BVS results indicate that Fe_4_O_5_ with a nominal average oxidation state of iron as Fe^2.5+^ tends to reach a state under pressure with more uniform charge distribution, implying an average oxidation state of Fe^2.5+^ in all iron chains.

**Fig. 7:**
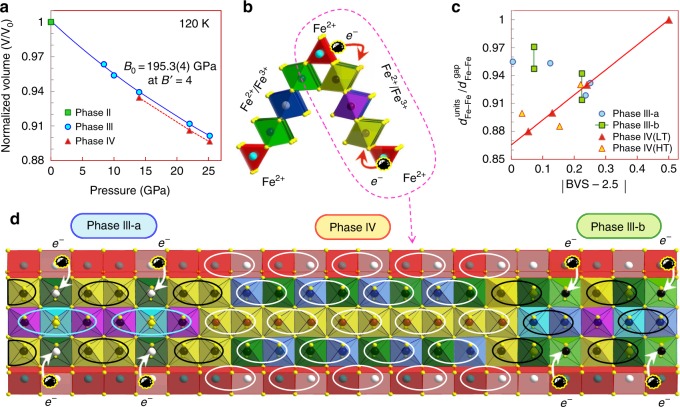
Pressure-driven Fe_4_O_5_-III → Fe_4_O_5_-IV transition. **a** Equation of state of Fe_4_O_5_ at 120 K showing a volume drop of ~0.5% at the Fe_4_O_5_-III → Fe_4_O_5_-IV transition. **b** Edge-shared diagonal ribbons of prisms and octahedra (seen in Figs. 4–6) provide channels for charge hopping between the prisms and octahedra. **c** Correlation between ratios of Fe–Fe distances in dimers and trimers to those in gaps between them $\left( {d_{{\mathrm{Fe}} - {\mathrm{Fe}}}^{{\mathrm{units}}}/d_{{\mathrm{Fe}} - {\mathrm{Fe}}}^{{\mathrm{gap}}}} \right)$ and absolute deviations of bond valence sums (BVS) values of iron in these chains from 2.5+. **d** Model demonstrating the coexistence of several charge-ordered phases in a single crystal of Fe_4_O_5_. The electron transfer from the prisms to octahedra leads to a short-range reorganization of ordering type

During the Fe_4_O_5_-III → Fe_4_O_5_-IV phase transition under increasing pressure, the crystal lattice shrinks noticeably along the dimerization direction. This leads to a lattice volume collapse by about 0.5% at 120 K (Fig. 7a). This volume collapse may be attributed to a more homogeneous charge distribution in the Fe_4_O_5_-IV phase, which should lead to a volume contraction. By fitting the third-order Birch–Murnaghan equation of state^37,38^ to the compression data of the Fe_4_O_5_-III phase at 120 K (Fig. 7a), we determine the unit cell volume as *V*_0_ = 1063.87 Å^3^ and the bulk modulus as *B*_0_ = 195.3 GPa for fixed *B*′_0_ = 4. We analyze the ratios of Fe–Fe bond lengths in the dimer units compared to those in the gaps between them $\left( {d_{{\mathrm{Fe}} - {\mathrm{Fe}}}^{{\mathrm{units}}}/d_{{\mathrm{Fe}} - {\mathrm{Fe}}}^{{\mathrm{gap}}}} \right)$ for Fe1, Fe2, and Fe3 chains, and values to be 0.93, 0.90, and 0.88, respectively (Supplementary Table 4). This difference in the $d_{{\mathrm{Fe}} - {\mathrm{Fe}}}^{{\mathrm{units}}}/d_{{\mathrm{Fe}} - {\mathrm{Fe}}}^{{\mathrm{gap}}}$ ratios may be referred to the above average BVS values of Fe1, Fe2, and Fe3 atoms, which should approximately correspond to their oxidation states. In fact, the shortest iron dimers are formed in the double chains of Fe3 octahedra with an average BVS value equal to +2.554, i.e., nearly optimal for the formation of Fe^2+^ –Fe^3+^ pairs. In contrast, the longest dimers are formed in Fe1 prismatic chains with an average BVS value of +2.26, which deviates the most from +2.5. Furthermore, we note that the above $d_{{\mathrm{Fe}} - {\mathrm{Fe}}}^{{\mathrm{units}}}/d_{{\mathrm{Fe}} - {\mathrm{Fe}}}^{{\mathrm{gap}}}$ ratios increase linearly with a deviation of their average BVS values from +2.5, tending to 1 for the limiting case of Fe^2+^ or Fe^3+^ (Fig. 7c). This regularity indicates that the resulting length of the dimers is determined by the balance of Fe^2+^ and Fe^3+^ cations in each linear chain.

The reduced distances between the free Fe3 atoms in the double-chains of Fe3 octahedra and the neighboring Fe1 atoms in the prisms, observed in both Fe_4_O_5_-III-a and Fe_4_O_5_-III-b structures (highlighted as dashed ellipsoids in Figs. 4d and 5d), appear to be the most suitable channels for hopping of electrons from Fe^2+^ ions occupying Fe1 prisms to the octahedral matrix. Only 1/3 of the Fe1 atoms are involved in these channels, and hence, if the electrons of prismatically-coordinated Fe^2+^ ions are not delocalized within the framework of these trigonal prismatic chains, the Fe1 atoms may be maximally charged up to +2.33 on average. The actual BVS values of the two Fe1 atoms are +2.23 and +2.29 (Fig. 6), which satisfy this constraint well. The coexistence of Fe_4_O_5_-III and Fe_4_O_5_-IV phases over an extended pressure range (Fig. 2) suggests that this phase transition is rather prolonged. Likely, it is caused by the gradual pressure-driven electron transfer from the trigonal prismatic chains to the octahedral ones. The Fe_4_O_5_-III-b structure comprises only dimers; hence having free iron cations in all Fe3 chains provides twice as many channels for the charge hopping compared to the Fe_4_O_5_-III-a structure.

As mentioned above, we observe that a noticeable fraction of the Fe_4_O_5_-IV phase persists upon heating to 293 K at 35 GPa (Fig. 2). To examine the possibility of the room-temperature phase transition Fe_4_O_5_-I → Fe_4_O_5_-IV, we compress a single crystal of Fe_4_O_5_ in a separate experiment to about 45 GPa, but we see no evidence for a phase transition. Previous works also did not find any structural phase transition in this range^12,39^. At the maximum pressure of 45 GPa, we laser-heat the sample up to 2000 °C, and indeed we observe a structural transition to the same dimerized Fe_4_O_5_-IV structure (Fig. 2, Table 1). The unusual thermodynamic stability of the Fe_4_O_5_-IV phase might be explained by the fact that both Fe_4_O_5_-III-a and Fe_4_O_5_-III-b low-temperature structures have the above-discussed Fe3–Fe1 channels for charge hopping (Figs. 4d and 5d), which should facilitate the Fe_4_O_5_-III → Fe_4_O_5_-IV transition; whereas a direct Fe_4_O_5_-I → Fe_4_O_5_-IV transition and its related redistribution of electrons between the prismatic and octahedral chains might be hindered at room temperature, but triggered at high temperatures. We find that average BVS values^36^ of the iron ions in the high-temperature Fe_4_O_5_-IV (HT) phase are nearly the same as those in the low-temperature Fe_4_O_5_-IV (LT) phase, namely, +2.24 (+2.32) for prismatic Fe1_1 (Fe1_2) atoms, and +2.653 and +2.534 for the iron in the single chains of Fe2 and double chains of Fe3 octahedra, respectively. Analysis of $d_{{\mathrm{Fe}} - {\mathrm{Fe}}}^{{\mathrm{units}}}/d_{{\mathrm{Fe}} - {\mathrm{Fe}}}^{{\mathrm{gap}}}$ ratios in the Fe_4_O_5_-IV (HT) structure shows that they do not follow the linear trend established for the Fe_4_O_5_-IV (LT) structure (Fig. 7c), and hence a distribution of Fe^2+^ and Fe^3+^ ions at least at the octahedral sites of Fe_4_O_5_-IV (HT) is more random than ordered. It can be that the transition mechanism at high temperature is different, and the dimerization observed could instead result from a Peierls transition^40^ rather than from the formation of electrically-bonded Fe^2+^–Fe^3+^ dimers with a shared electron.

### Low-temperature high-pressure phase diagram of Fe_4_O_5_

We find the average BVS values of prismatic Fe1 atoms in Fe_4_O_5_-I, Fe_4_O_5_-III-a, Fe_4_O_5_-III-b, and Fe_4_O_5_-IV to be +2.016, +2.11, +2.13, and +2.26, respectively (Figs. 4–6). As discussed above, the average BVS value of +2.26 for Fe1 atoms in Fe_4_O_5_-IV, together with the overall structural dimerization including these Fe1 chains, unambiguously confirm the mixed-valent nature of the iron ions at the prismatic sites in this structure. The minor deviations of the BVS values from +2 for Fe1 atoms in the Fe_4_O_5_-III-a and Fe_4_O_5_-III-b phases do not allow us either to conclude a small shift in the oxidation state of these ions toward +3 or to rule that out. The co-existence of Fe_4_O_5_-III and Fe_4_O_5_-IV phases for an extended pressure range (Fig. 2) hints that this transition may follow the electron transfer between the octahedral and prismatic chains. This leads to a short-order reorganization of the charge-ordering pattern and the formation of inclusions of the novel structural order. Comparing the different charge-ordered patterns in Fe_4_O_5_-II^32^, Fe_4_O_5_-III-a, Fe_4_O_5_-III-b, and Fe_4_O_5_-IV (Figs. 4–6), we conclude that the average oxidation state of octahedrally-coordinated cations predetermines which ordering type would be optimal. For example, in order to form short trimers, the average oxidation state of iron should be ~+2.7 (Fig. 7c). A moderate decrease in this BVS value leads to the formation of more loosely-bonded long trimers and dimers. For BVS values tending toward +2.5, a closely-packed dimeric order becomes preferable (Fig. 6). Thus, the average oxidation state of iron ions at the octahedral chains apparently pre-determines an optimal charge-ordering scheme. One can expect, for example in the newly-discovered Fe_7_O_9_ and Fe_5_O_6_ crystallizing in similar structures^14,22^, that the former with a nominal oxidation state of the octahedral ions of +2.8 at ambient pressure is prone to the formation of exclusively trimers; likewise, the latter in which their oxidation state is +2.5 is prone to dimer formation only. A pressure-stimulated electron transfer from the prismatic to octahedral chains can occur inhomogeneously in the bulk of the Fe_4_O_5_ crystal and this can result in the formation of an anomalous charge-ordering pattern combining different co-existing ordering schemes (Fig. 7d).

The process of electron transfer in Fe_4_O_5_ from the trigonal prismatic to the octahedral iron chains towards charge equalization is perhaps not complete by 40 GPa, and with further pressurization the average BVS values of all iron cations can probably approach +2.5 even more closely. However, this could hardly change the dimeric charge-ordering pattern of Fe_4_O_5_-IV (Fig. 6), unless the material was to become metallic and the charge-ordering state suppressed; although the lattice symmetry may be sensitive to the charge balance. We propose that such electron transfer processes under pressure could also occur in other iron oxides crystallizing in kindred lattices, like the above-mentioned Fe_5_O_6_^14^ and Fe_7_O_9_^22^. However, for the ambient-pressure cubic spinel phase of magnetite with inverse electronic configuration in which tetrahedral sites are filled by Fe^3+^ ions and hence are already maximally charged, the possibility of an opposite electron transfer under pressure, from tetrahedral sites to the octahedral network has been suggested^41^. However, this conjecture was not in line with earlier work^42^, and was not confirmed in subsequent studies^43–46^. It was established for magnetite that its charge-ordered phase may be suppressed by applied pressure of about 8 GPa^47,48^. Thus, the behavior of Fe_4_O_5_ is remarkably different from that of magnetite and demonstrates new perspectives for charge-ordered states in iron-rich oxides. At the moment, we cannot unambiguously ascertain the driving forces of the phase transitions observed in Fe_4_O_5_ both at ambient and high pressures. In previous work investigating the Verwey transition in magnetite, it was determined that the intersite Coulomb interactions between the 3*d* electrons of the Fe ions alone, as well as phonon-driven lattice instability, could hardly stimulate this transition^49,50^. A more complex scenario, however, in which the strong electron correlations enhance the electron–phonon interactions and simultaneously reduce the mobility of the minority-spin *t*_2*g*_ electrons of Fe^2+^ ions, increasing their tendency towards localization, could be an indication that electron–phonon interactions may be a driving force of the Verwey transition^49,50^. The phase transitions in Fe_4_O_5_ could have similar or even more complex scenarios involving the charge, lattice, spin, and orbital degrees of freedom.

### Mössbauer spectroscopy of Fe_4_O_5_ at low temperature under high pressure

**Fig. 8:**
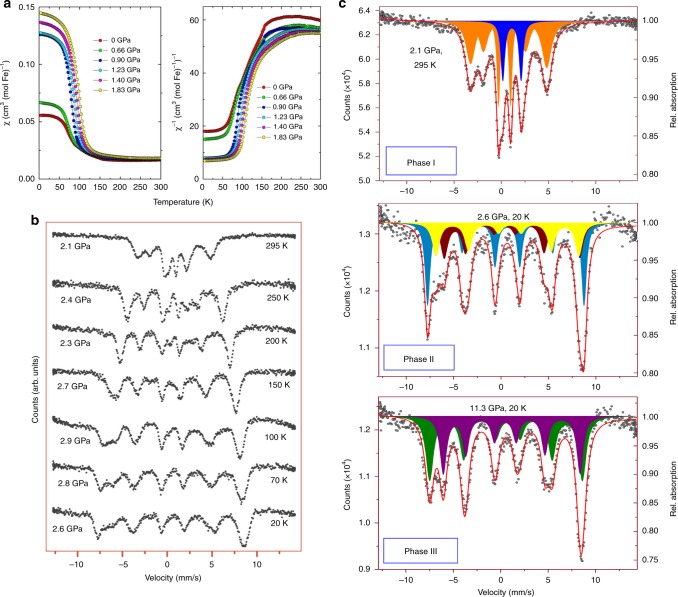
Magnetic susceptibility and Mössbauer spectra of Fe_4_O_5_. **a** Temperature dependence of magnetic susceptibility and inverse magnetic susceptibility measured at different hydrostatic pressures in a field of 0.5  T under field cooling. **b** Evolution of Mössbauer spectra upon cooling at about 2–3 GPa. Broadening of the first (low-velocity) line of the sextet starting at 150 K indicates charge ordering. **c** Representative Mössbauer spectra of different phases of Fe_4_O_5_ collected at different pressures and temperatures. The spectrum of phase I can be refined by superposition of a paramagnetic doublet (blue) assigned to iron in the trigonal prisms and a magnetic sextet (orange) assigned to Fe in the octahedra in a mixed-valence state. The spectra of phases II and III exhibit a complex distribution of hyperfine fields and can be refined by superposition of sextets

At ambient conditions, the Mössbauer spectra of Fe_4_O_5_ can be well fitted by the superposition of a magnetic sextet and a paramagnetic doublet with relative areas of 80(2)% and 20(2)%, respectively (Fig. 8c). The presence of the sextet component indicates the existence of magnetic order at room temperature. In the Fe_4_O_5_ crystal, 25% of iron cations occupy the trigonal prisms, and 75% have octahedral coordination (Fig. 1a). Hence, we can assign the doublet to prismatic Fe1 atoms, and likewise the sextet to octahedral Fe2 and Fe3 atoms. We determine the hyperfine parameters of these components. For example, for the spectrum collected at 295 K and 2.1 GPa, we determine the center shift of the doublet to be *δ*_CS_ = 1.125(12) mm/s and the quadrupole splitting to be ∆ = 1.93(2) mm/s. For the sextet, we find the center shift to be *δ*_CS_ = 0.568(15) mm/s, the quadrupole shift to be *ε* = 0.21(1) mm/s, and the hyperfine magnetic field to be *H*_hf_ = 24.96(13) T. The center shift of the Mössbauer spectral components depends primarily on the oxidation state of iron in a linear-like manner. We compare these center shifts with those reported for other iron oxides, e.g., *δ*_CS_ = 0.36 mm/s for octahedral Fe^3+^ ions in hematite (α-Fe_2_O_3_)^51^ and *δ*_CS_ = 0.67 mm/s for octahedral mixed-valent Fe^2.5+^ ions in magnetite^51^. The linear trend based on these data suggests that octahedrally-coordinated Fe^2+^ ions should exhibit center shifts around *δ*_CS_ = 0.98 mm/s; likewise the above-determined value of *δ*_CS_ = 0.568 mm/s for the magnetic sextet in Fe_4_O_5_ should correspond to an oxidation state of Fe^2.67+^, in excellent agreement with the BVS results (Fig. 1a). The large value of *δ*_CS_ = 1.125(12) mm/s for the Fe1 doublet unambiguously corresponds to Fe^2+^, in line with both BVS estimations (Fig. 1a) and earlier data for ferrous-iron compounds^51^.

We note that the line widths of the sextet are roughly twice as large as those of the doublet (1.1 vs 0.5 mm/s) (Fig. 8c). This broadening results from electron exchange between the Fe^2+^ and Fe^3+^ cations occupying the octahedral sites; a similar effect was also observed in magnetite^52^. Upon cooling, the doublet component loses intensity and completely disappears around 150 K (Fig. 8b). Hence, Fe1 atoms occupying the oxygen prisms become magnetically ordered, and their contribution to the spectra largely overlaps with the stronger signal of Fe2 and Fe3 atoms. The Mössbauer spectra demonstrate a pronounced broadening around 150 K (Fig. 8b), where a charge ordering in Fe_4_O_5_ was observed^32^.

For magnetite, it has been documented that charge ordering below the Verwey transition at 120 K leads to the appearance of many closely-overlapping magnetic sextets which can be resolved only under special conditions, such as single-crystal measurements in magnetic fields^52,53^. Here, we collect spectra from a polycrystalline Fe_4_O_5_ sample and, together with other factors like potential phase co-existence (Fig. 2), this impedes an unambiguous fitting of the Mössbauer spectra at low temperatures. A simple single-sextet analysis of the spectra shows that the quadrupole shift has a discontinuity in its temperature dependence between 150 and 100 K (Supplementary Figure 2b and Supplementary Table 5). This feature is likely linked to changes in the magnetic properties (kink in inverse magnetic susceptibility in Fig. 7a).

The Mössbauer spectra collected in the charge-ordered states appear to be a superposition of several sextets. For instance, the spectrum acquired at 20 K and 2.6 GPa, i.e., in the stability region of the incommensurately-modulated Fe_4_O_5_-II phase with an infinite number of different environments for iron (Fig. 2), can be reasonably well described by three sextets (Fig. 8c). The center shifts of these sextets are about 0.6–0.85 mm/s, i.e., between *δ*_CS_ = 0.36 mm/s for Fe^3+^ ions in hematite^51^ and 0.98 mm/s estimated above for octahedral Fe^2+^ atoms, but at the same time quite far from both. We therefore conclude that in the charge-ordered phases of Fe_4_O_5_, the oxidation states of the octahedrally-coordinated iron did not split into Fe^2+^ and Fe^3+^ components, and hence, these ions are characterized by stable non-integer oxidation states. This finding is in line with the earlier conjecture^4,32^ that the minority-spin *t*_2*g*_ electron of Fe^2+^ ions is shared between all ions involved in the formation of either trimers or dimers.

We do not observe any noticeable changes in the spectra with pressure up to 8 GPa at low temperatures. This fact indicates that Fe_4_O_5_-II and Fe_4_O_5_-III charge-ordered phases are not readily distinguishable by Mössbauer spectroscopy (Figs. 2 and 8c). With further compression to 16.3 GPa across the beginning of the Fe_4_O_5_-III → Fe_4_O_5_-IV transition (Fig. 2), the hyperfine field distribution changes to an apparent bimodal form (Supplementary Figure 2c). We fit these spectra by a superposition of two sextets (Fig. 8c), and for example, at 11.3 GPa and 20 K in Fe_4_O_5_-III phase, their center shifts are *δ*_CS_ = 0.66(3) and 0.82(3) mm/s. Taking into account the second-order Doppler shift (we use the value of 0.11 mm/s for hematite)^54^ and disregarding a possible pressure correction for the *δ*_CS_ values, we estimate the average oxidation states of iron linked to the green and purple sextets to be +2.7 and +2.45, respectively (Fig. 8c). Therefore, this case also unambiguously demonstrates that the oxidation states of the octahedrally-coordinated iron in Fe_4_O_5_ do not split into integer Fe^2+^ and Fe^3+^ components, even at 20 K. These +2.7 and +2.45 values correspond well to the average BVS values for the iron cations occupying the single chains of Fe2 and double chains of Fe3 octahedra, respectively (Figs. 4 and 5).

### Magnetic properties of Fe_4_O_5_

The magnetization data for Fe_4_O_5_ collected at ambient pressure (Fig. 8a) are similar to those reported earlier^32^, but show slightly smaller absolute values of susceptibility. In this work, we carry out measurements on several single crystals of Fe_4_O_5_, and likely, this discrepancy may be related to a minor ferromagnetic impurity which can potentially be present in the large polycrystalline sample examined earlier^32^. There are two main features of the magnetization data: (i) the transformation between canted and collinear magnetic ordering (around 90 K at ambient pressure), and (ii) charge ordering that manifests itself by a kink in the 1/*χ* curve (around 150 K at ambient pressure) (Fig. 8a). Both features are observed up to 1.83 GPa, the maximum pressure of our measurement, and shift toward higher temperature upon compression. The size of the canted moment increases abruptly, with a large change between 0.66 and 0.90 GPa and weak changes below or above this range. Given the observation of the Fe_4_O_5_-III phase at 200 K and 2 GPa (Fig. 2), we conclude that our magnetization data extend well into its stability range, and hence, this abrupt change in the moment likely results from the phase transformation. Moreover, the kink in the 1/*χ* curves shifts to higher temperatures (Fig. 8a), in agreement with the positive slope of the Fe_4_O_5_-I → Fe_4_O_5_-III phase boundary. Above 0.90 GPa, the kink is strongly smeared out, probably because of more sluggish charge-ordering processes at the Fe_4_O_5_-I → Fe_4_O_5_-III phase transition, compared to Fe_4_O_5_-I → Fe_4_O_5_-II^32^.

The magnetization data reveal close similarities between Fe_4_O_5_-II and Fe_4_O_5_-III. Both support the formation of a canted state at low temperatures. Moreover, both charge-ordered phases emerge from the same collinear magnetic order that sets in around 320 K at ambient pressure^32^. As seen from the Mössbauer spectra (Fig. 8b), the initial magnetic ordering remains above room temperature at these pressures. As established earlier^32^, a ferromagnetic spin alignment along the *a*-direction (in the coordinate framework of Fe_4_O_5_-I) is essential for the formation of dimers and trimers in the charge-ordered state^32^, and hence magnetic ordering is a key prerequisite of the charge ordering. The same type of magnetic order would even support dimer formation in Fe_4_O_5_-IV. This demonstrates that this magnetic ordering can produce different charge-ordered states in a single structural framework.

### Conclusions

We determined the low-temperature high-pressure phase diagram of Fe_4_O_5_ using single crystal X-ray diffraction and Mössbauer spectroscopy and by measurement of magnetic properties. We found two novel crystal structures of Fe_4_O_5_ and observed the extended regions of their co-existence in the phase diagram. A dramatic change in the charge-ordering pattern in the second high-pressure phase was attributed to electron hopping from the octahedral to the prismatic iron chains. We propose that the average oxidation state of the iron cations in oxides of this family can pre-determine a charge-ordering pattern. Thus, Fe_4_O_5_ demonstrates that the charge-ordering pattern can be changed by applied pressure or stress, and two or more charge-ordered phases can co-exist with each other inside one single crystal. Our work highlights the complexity of charge-ordering processes in iron-based and other transition metal oxides, but simultaneously it suggests a clue to these phenomena.

## Methods

### Sample preparation and characterization

The samples of Fe_4_O_5_ were synthesized in a 1200-tonne Multi-Anvil Press at BGI^55^ at HP-HT conditions from stoichiometric mixtures of fine powders of Fe_3_O_4_ (Aldrich, 99.99% purity) and Fe (Aldrich, 99.999% purity). The syntheses were performed at pressures of 14 GPa over several hours. Polycrystalline samples were fabricated at synthesis temperatures of about 1100–1200 °C, while higher temperatures (1350–1450 °C) enabled the growth of high-quality single crystals with linear sizes of 20–200 µm^32^. We employed a standard assembly including a Re cylindrical sample capsule, a LaCrO_3_ heater, a W3Re/W25Re thermocouple, and other components packed inside an octahedron made of 5% Cr_2_O_3_-doped MgO^56,57^. The procedure was similar to that described in previous work^58,59^. The chemical composition of the samples was verified by means of scanning electron microscopy (SEM) using a LEO-1530 instrument and by microprobe analysis using a JEOL JXA-8200 electron microscope. The crystal structure of the samples was determined by means of single crystal and powder X-ray diffraction using a high-brilliance Rigaku diffractometer (Mo *K*_α_ radiation, *λ* = 0.7108 Å).

### Single crystal X-ray diffraction under pressure

We selected high-quality single crystals of Fe_4_O_5_ and loaded them into symmetric membrane diamond anvil cells (DACs) equipped with Boehler-Almax diamonds that enabled X-ray diffraction images to be collected over the widest range of angles. We employed three DACs with diamond anvil culet sizes of 400 and 300 µm. Together with the sample in the same cell, we loaded a ruby sphere and a chip of gold that was used for pressure determination (Fig. 2). An additional ruby sphere was placed on the outer surface of one diamond anvil to monitor the reference of its *R*_1_ line at low temperature and ambient pressure. All of the DACs were filled with Ne pressure-transmitting medium. In total, we carried out three high-pressure single crystal X-ray diffraction experiments. The first one served as an initial scanning of the low-temperature phase diagram of Fe_4_O_5_, and it was performed at the ID27 beamline of the European Synchrotron Radiation Facility (ESRF, Grenoble, France) with a wavelength of *λ* = 0.3738 Å. The second and third runs involved more detailed investigations, and they were accomplished in a cold-finger He cryostat on the P02.2 beamline at the Deutsches Elektronen-Synchrotron (DESY, Hamburg, Germany) using a wavelength of *λ* = 0.2887 Å^60^. Additionally, we carried out a room temperature compression experiment with laser heating at high pressures on beamline ID09a at ESRF (*λ* = 0.41513 Å). At each (*P*,*T*) point on the phase diagram, we acquired single-crystal X-ray diffraction images upon continuous rotation of the cell with the sample around the vertical *ω*-axis with a step of Δ*ω* = 0.5° and an exposure time of *t* = 0.5 s/frame. The diffraction data were collected by a Perkin Elmer XRD1621 detector. We analyzed these data with CrysAlisPro software, and solved the crystal structures using JANA2006 software^61^.

We analyze the Fe–O bond lengths of all iron cations in the different crystal structures of Fe_4_O_5_ using a common BVS method^36^. In this method, a bond valence is determined as (*S*_*ij*_ = exp[(*R*_*ij*_ − *d*_*ij*_)/*b*_0_], where *d*_*ij*_ is the distance between atoms *i* and *j*, *R*_*ij*_ is the bond valence parameter (empirically determined distance for this cation-anion pair), and *b*_0_ is an empirical parameter about 0.37 Å), and then, a BVS value of a cation is determined as a sum of individual bond valences $\left( {V_i = \mathop {\sum}\nolimits_j {s_{ij}} } \right)$^36^. In these calculations, we used *b*_0_=0.37 Å and the bond-valence parameters *R*_*ij*_ determined at ambient conditions for Fe^2+^–O and Fe^3+^–O bonds as 1.734 and 1.759 Å, respectively^36^. Using literature data from the equation of state of hematite (α-Fe_2_O_3_) determined from single crystal X-ray diffraction experiments up to 25 GPa^62^, we estimated a pressure dependence of the bond-parameter *R*_*ij*_ for the Fe^3+^–O bonds, and applied these values in the BVS estimations as well. Since the total cation charge in the formula unit (+10) should be conserved at all pressures and temperatures, the calculated nominal BVS values were accordingly renormalized to meet this requirement. After performing these procedures, the calculations using different starting *R*_*ij*_ values gave identical results within experimental uncertainty.

### Mössbauer spectroscopy under pressure

For Mössbauer spectroscopic examination over a wide pressure–temperature range, we synthesized a polycrystalline sample of 20% ^57^Fe-enriched Fe_4_O_5_. We also utilized a membrane DAC with diamond anvil culet sizes of 400 µm. The DAC was fixed inside a cryostat. We collected synchrotron Mössbauer source (SMS)^63^ spectra on the Nuclear Resonance beamline ID18 at ESRF^64^. The SMS is based on a nuclear resonant monochromator employing pure nuclear reflections of an iron borate (^57^FeBO_3_) crystal. The source provides ^57^Fe resonant radiation at 14.4 keV within a bandwidth of 15 neV, which is tunable in energy over a range of ~±0.6 meV^63^. The beam of gamma-radiation emitted by the SMS was focused to a 10 × 15 µm^2^ spot size. The velocity scale was calibrated relative to a natural α-Fe foil of 25 μm thickness. The center shift values are given relative to α-Fe. We monitored the width and the absolute position of the isomer shift of the source line before and after each measurement using a K_2_Mg^57^Fe(CN)_6_ reference single line absorber.

### Magnetic measurements under pressure

The bulk magnetization measurements under hydrostatic pressure^65,66^ were performed in a CuBe pressure cell placed inside a Quantum Design MPMS 5S SQUID magnetometer. Daphne 7373 oil was used as a pressure-transmitting medium. One small piece of lead (~0.1 mg) was placed together with the sample inside the pressure cell, while another piece (~0.1 mg) was placed outside the pressure cell. Under pressure, the superconducting transition temperature of the inner piece decreases. The difference between the superconducting transition temperatures of the two lead samples determines the pressure value inside the pressure cell at low temperatures. Several small single crystals of Fe_4_O_5_ with a total mass of ~0.6 mg were placed into a gasket of the pressure cell along with the aforementioned piece of lead. The empty cell background data was subtracted^65^ using an automatic background subtraction (ABS) procedure. Field-cooling measurements of the lead and Fe_4_O_5_ samples were performed in fields of 2 mT and 0.5 T, respectively, from 300 K down to 4 K.

## Electronic supplementary material

Supplementary Information

Description of Additional Supplementary Files

Supplementary Data 1

Supplementary Data 2

Supplementary Data 3

Supplementary Data 4

Supplementary Data 5

## Data Availability

The X-ray crystallographic information files (CIFs) for structures that support the findings of this study have been deposited at the Inorganic Crystal Structure Database (ICSD) with accession codes 434152, 434153, 434154, 434155, and 434156 (http://www2.fiz-karlsruhe.de/icsd_home.html). The authors declare that all other data supporting the findings of this study are available within the article and Supplementary Information files, and also are available from the corresponding author upon reasonable request.
