# Supplementary material for: Pressure tuning of charge ordering in iron oxide
Source: Nat Commun. 2018 Oct 8;9:4142. doi: 10.1038/s41467-018-06457-x (PMC6175922; doi:10.1038/s41467-018-06457-x)
Supplement: Supplementary file 1 — Supplementary Information [file 41467_2018_6457_MOESM1_ESM.pdf]

Supplementary Information

Pressure tuning of charge ordering in iron oxide

Ovsyannikov et al.

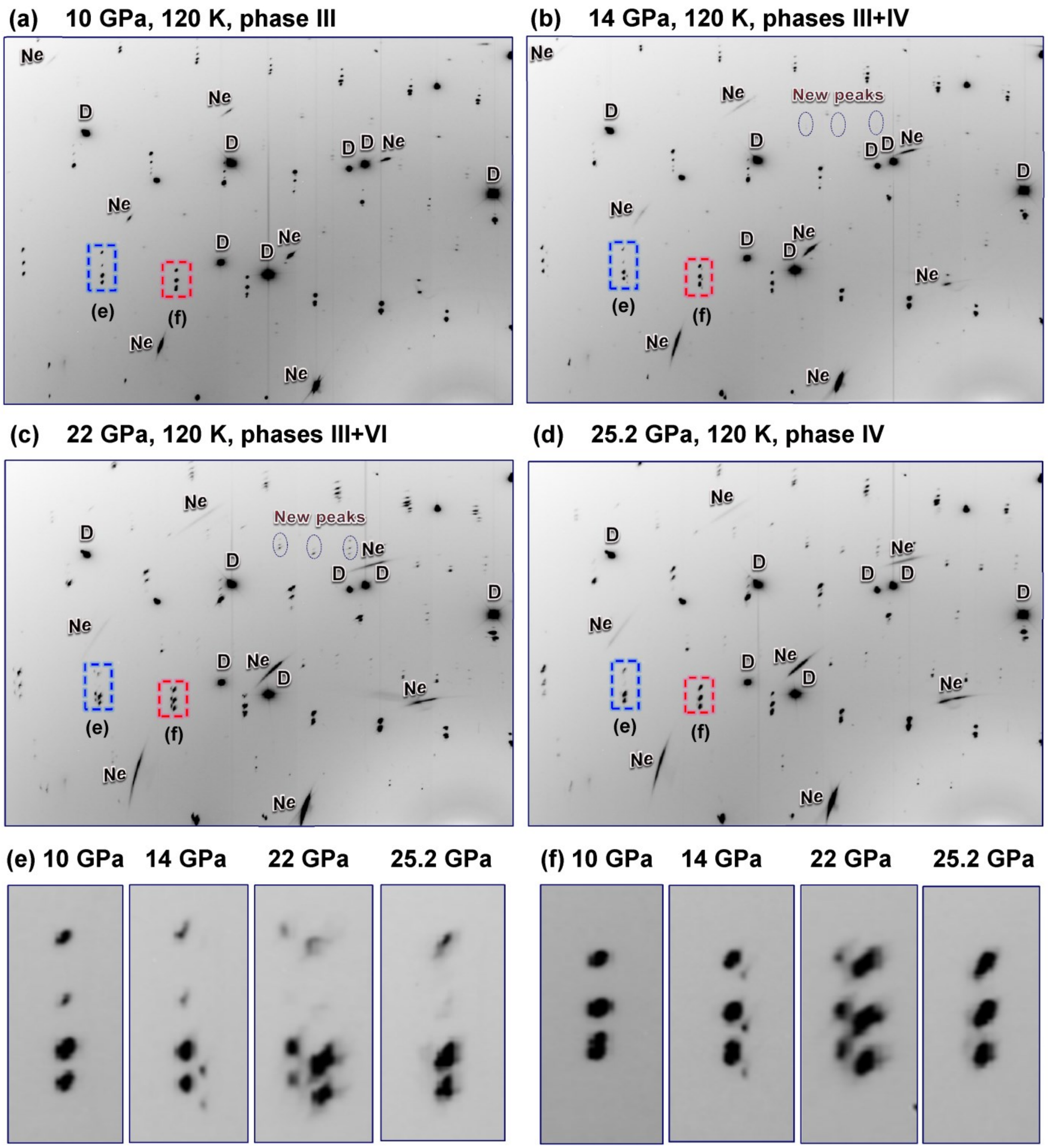

Supplementary Figure 1. Examples of wide-rotation X-ray diffraction images of Fe_4O_5. These X-ray diffraction images of Fe_4O_5 were collected at 120 K across the Fe_4O_5-III → Fe_4O_5-IV transition. The four upper plots show roughly the quarters of the full X-ray diffraction images collected at different pressures. Labels *D* and *Ne* indicate the large reflections originating from the diamond anvils and solidified neon pressure medium, respectively. The remaining structural reflections are from Fe_4O_5 (the stronger reflections are basic, while the weaker ones are satellite). The two lower panels, (***e***) and (***f***), are combinations of selected magnified parts highlighted in the upper plots (***a-d***). They show that the Fe_4O_5-III → Fe_4O_5-IV transition starts somewhat above 10 GPa and is nearly completed at 25 GPa.

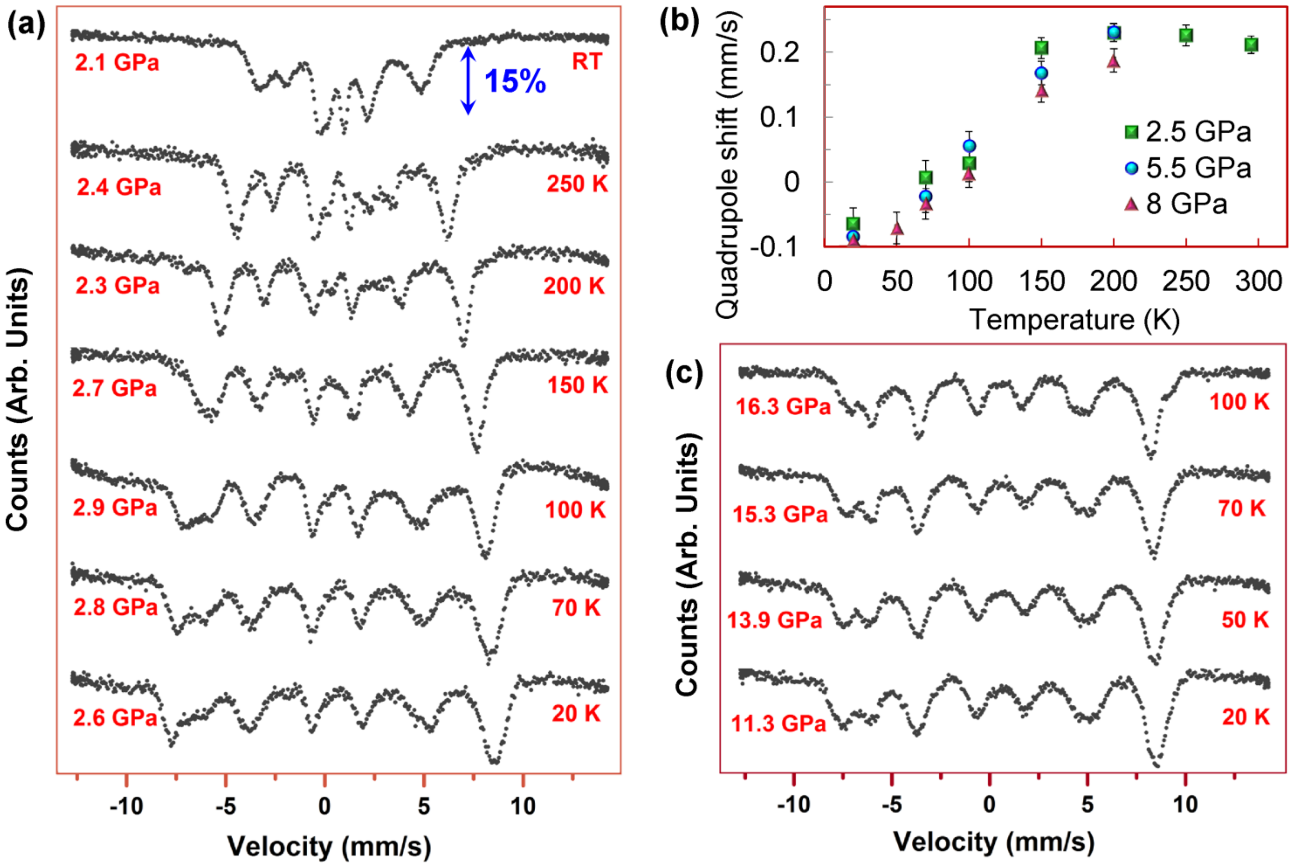

Supplementary Figure 2. Evolution of Mössbauer spectra of Fe_4O_5 and of quadrupole shift. (***a***) Evolution of spectra upon cooling at about 2-3 GPa. Broadening of the first (low-velocity) line of the sextet starting at 150 K indicates charge ordering. (***b***) Temperature dependencies of quadrupole shift determined in single-sextet model at different pressures showing distinct a discontinuity between 150 and 100 K. This feature can indicate a magnetic transition linked to reorientation of magnetic moments. (***c***) Evolution of spectra across the beginning of the Fe_4O_5-III → Fe_4O_5-IV transition, showing a variation in the first sextet line.

Supplementary Table 1. Results of testing of different models for the crystal structure of the phase III (Fe_4O_5-III) generated by the distortion mode analysis using the ISODISTORT program.

Distortion	Lattice parameters	Space group		R_1, %
Σ_1<br>P1 (a,0)	a=8.62098 Å<br>b=9.69400 Å<br>c=12.41160 Å	*Cmcm*	basis={(3,0,0),(0,1,0),(0,0,1)},<br>origin=(0,0,0)	18.32
Σ_1<br>C1 (a, b)	a=12.41160Å<br>b=9.69400 Å<br>c=8.62098 Å	*Ama*2	basis={(0,0,1),(0,1,0),(-3,0,0)},<br>origin=(0,0,0)	15.28
**Σ_2**<br>**P1 (a,0)**	**a=8.62098 Å**<br>**b=9.69400 Å**<br>**c=12.41160 Å**	***C*222$_1$**	**basis={(3,0,0),(0,1,0),(0,0,1)},**<br>**origin=(0,0,0)**	**5.31**
**Σ_2**<br>**P2 (0,a)**	**a=9.69400 Å**<br>**b=8.62098 Å**<br>**c=12.41160 Å**	***C*2/*m***	**basis={(0,-1,0),(3,0,0),(0,0,1)},**<br>**origin=(0,0,0)**	**5.84**
**Σ_2**<br>**C1(a,b)**	**a=9.69400 Å**<br>**b=8.62098 Å**<br>**c=12.41160 Å**	***C*2**	**basis={(0,-1,0),(3,0,0),(0,0,1)},**<br>**origin=(0,0,0)**	**5.09**
Σ_3<br>*P*1 (a,0)	a=8.62098 Å<br>b=9.69400 Å<br>c=12.41160 Å	*C*2/*c*	basis={(-3,0,0),(0,-1,0),(0,0,1)}<br>origin=(0,0,0)	17.39
Σ_3<br>*P*2 (0,a)	a=8.62098 Å<br>b=9.69400 Å<br>c=12.41160 Å	*Cmc*2$_1$	basis={(3,0,0),(0,1,0),(0,0,1)},<br>origin=(0,0,0)	12.29
Σ_3<br>C1 (a,b)	a=8.62098 Å<br>b=9.69400 Å<br>c=12.41160 Å	*Cc*	basis={(-3,0,0),(0,-1,0),(0,0,1)},<br>origin=(0,0,0)	10.99
Σ_4<br>P1 (0,a)	a=6.48643 Å<br>b=12.41160 Å<br>c=6.48643 Å<br>β =96.70591°	*P*2$_1$/*m*	basis={(3/2,1/2,0),(0,0,1),(3/2,-<br>1/2,0)}, origin=(0,0,0)	18.05
Σ_4<br>P2 (a,0)	a=12.41160 Å<br>b=8.62098 Å<br>c=9.69400 Å	*Amm*2	basis={(0,0,1),(3,0,0),(0,1,0)},<br>origin=(0,0,1/4)	11.91
Σ_4<br>C1 (a,b)	a=6.48643 Å<br>b=12.41160 Å<br>c=6.48643 Å<br>β=96.70591°	*Pm*	basis={(3/2,1/2,0),(0,0,1),(3/2,-<br>1/2,0)}, origin=(0,0,1/4)	11.71

Supplementary Table 2. Details of the crystal structure of the original phase and of the HP-LT structures of Fe_4O_5–III and details of their crystal structure refinements from single crystal X-ray diffraction data*.

Phases:	Fe_4O_5-I	Fe_4O_5-III-a	Fe_4O_5-III-b
	Details of crystal structures		
Pressure (GPa)	ambient	11.7	11.7
Temperature (K)	293	180	180
Crystal system	Orthorhombic	Monoclinic	Orthorhombic
Space group (No.)	*Cmcm* (No. 63)	*C*2/*m* (No. 12)	$C222_1$ (No. 20)
Lattice parameter, *a* (Å)	2.89200(5)	9.675(4)	8.4492(3)
Lattice parameter, *b* (Å)	9.7979(2)	8.4493(3)	9.6750(4)
Lattice parameter, *c* (Å)	12.583(2)	12.328(7)	12.328(7)
β (°)		90.0(1)	
Unit cell volume, V (Å^3)	356.54 (7)	1007.8 (6)	1007.7 (6)
Z	4	12	12
Calculated density (g/cm^3)	5.65138	5.99868	5.99875
	Details of crystal structure refinement		
Absorption correction, T_{min}, T_{max}	0.693, 1.000	0.591, 1.000	0.591, 1.000
Number of measured, independent and observed [$I > 3\sigma(I)$] reflections	1051, 285, 279	1613, 791, 578	1613, 1029, 735
R_{int}	0.028	0.021	0.040
$(\sin\theta/\lambda)_{max}$ (Å^{-1})	0.986	1.010	1.002
$R[F^2 > 2\sigma(F^2)]$, $wR(F^2)$, S	0.032, 0.039, 2.63	0.032, 0.048, 2.07	0.030, 0.041, 1.40
Number of reflections	285	791	1029
Number of parameters	32	100	88
$\Delta\rho_{max}$, $\Delta\rho_{min}$ (e·Å^{-3})	1.04, -1.23	0.83, -0.85	0.80, -0.75
	The shortest Fe-Fe distances in the prismatic and octahedral chains		
The shortest Fe-Fe distances, Å	2.8920 (Fe1–Fe1)	2.8176 (Fe1_1–Fe1_2)	2.8189 (Fe1_1–Fe1_2)
	2.8920 (Fe2–Fe2)	2.8143 (Fe1_2–Fe1_2)	2.8118 (Fe1_2–Fe1_2)
	2.8920 (Fe3–Fe3)	2.8175 (Fe1_1–Fe3_1)	2.8193 (Fe1_2–Fe3_1)
		2.8948 (Fe2_1–Fe2_2)	2.6777 (Fe2_1–Fe2_2)
		2.6597 (Fe2_2–Fe2_2)	2.9298 (Fe2_1–Fe2_3)
		2.7497 (Fe2_3–Fe2_4)	2.8416 (Fe2_2–Fe2_3)
		2.9500 (Fe2_4–Fe2_4)	2.8909 (Fe3_1–Fe3_2)
		2.7732 (Fe3_1–Fe3_2)	2.7387 (Fe3_1–Fe3_3)
		2.9039 (Fe3_2–Fe3_2)	2.8205 (Fe3_2–Fe3_3)
		2.8611 (Fe3_3–Fe3_4)	
		2.7279 (Fe3_4–Fe3_4)	

* This study was carried out at the P02.2 beamline at DESY (Hamburg) using a wavelength of λ = 0.2887Å

Supplementary Table 3. Atomic coordinates for the original and HP-LT phases of Fe_4O_5.

Atom	Site	x/a	y/b	z/c
Fe_4O_5-I, ambient conditions				
Fe1	4c	0	0.50561(5)	0.25
Fe2	4a	0	0	0
Fe3	8f	0	0.26036(4)	0.11754(7)
O1	4c	0	0.1603(2)	0.25
O2	8f	0	0.35895(17)	0.5444(4)
O3	8f	0	0.09275(17)	0.6422(3)
Fe_4O_5-III-b, 11.7 GPa, 180 K				
Fe1_1	4b	0	0.50382(12)	0.25
Fe1_2	8c	0.16638(6)	0.00417(7)	0.24851(9)
Fe2_1	4a	-0.00991(15)	0	0
Fe2_2	4a	0.67317(19)	0	0
Fe2_3	4a	0.33685(17)	0	0
Fe3_1	8c	0.00597(11)	0.26202(5)	0.1177(2)
Fe3_2	8c	0.66383(11)	0.26411(4)	0.11889(16)
Fe3_3	8c	0.33008(12)	0.25900(4)	0.11639(15)
O1	4b	0	0.1602(4)	0.25
O2	8c	0.3354(3)	0.1599(3)	0.2495(5)
O3	8c	-0.0035(4)	0.3602(2)	0.5428(9)
O4	8c	0.6647(5)	0.3534(2)	0.5466(6)
O5	8c	0.3331(5)	0.3629(2)	0.5441(6)
O6	8c	0.0015(6)	0.0896(2)	0.6448(10)
O7	8c	0.6660(6)	0.09005(19)	0.6424(6)
O8	8c	0.3316(6)	0.08861(19)	0.6435(6)

Atom	Site	x/a	y/b	z/c
Fe_4O_5-III-a, 11.7 GPa, 180 K				
Fe1_1	4i	0.49580(8)	0	0.2512(2)
Fe1_2	8j	0.49598(7)	0.33346(5)	0.24930(18)
Fe2_1	2a	0	0	0
Fe2_2	4g	0	0.34261(9)	0
Fe2_3	2c	0	0	0.5
Fe2_4	4h	0	0.32543(8)	0.5
Fe3_1	4i	0.74140(9)	0	0.1165(2)
Fe3_2	8j	0.73682(7)	0.32816(6)	0.11842(18)
Fe3_3	4i	0.73540(9)	0	0.3811(2)
Fe3_4	8j	0.73966(7)	0.33857(6)	0.38280(18)
O1	4i	0.8412(4)	0	0.2527(12)
O2	8j	0.8402(4)	0.3331(3)	0.2519(10)
O3	4i	0.6358(4)	0	0.5448(11)
O4	8j	0.6433(3)	0.3347(3)	0.5451(9)
O5	4i	0.6473(5)	0	-0.0465(12)
O6	8j	0.6386(3)	0.3320(3)	-0.0425(9)
O7	4i	0.9110(4)	0	0.6431(11)
O8	8j	0.9093(3)	0.3316(2)	0.6415(9)
O9	4i	0.9098(4)	0	0.8559(11)
O10	8j	0.9117(3)	0.3347(3)	0.8566(9)

Supplementary Table 4. Details of the crystal structure of Fe_4O_5-IV and its refinement from single crystal X-ray diffraction data*. The labels LT and HT denote the low-temperature or high-temperature (after laser heating) conditions of its synthesis, respectively.

Phases:	Fe_4O_5-IV (LT)*	Fe_4O_5-IV (HT)**
	Details of crystal structures	
Pressure (GPa)	25.2	48
Temperature (K)	120	296
Crystal system,	Monoclinic	Monoclinic
Space group (No.)	$P2_1/m$ (No. 11)	$P2_1/m$ (No. 11)
Lattice parameter, *a* (Å)	5.0145(10)	4.9408(10)
Lattice parameter, *b* (Å)	12.1155(18)	11.7880(18)
Lattice parameter, *c* (Å)	5.4282(4)	5.3287(4)
β (°)	105.582(11)	105.320(11)
Unit cell volume, *V* ($Å^3$)	317.66(8)	299.33(8)
Z	4	4
Calculated density (g/cm^3)	6.34361	6.73215
	Details of crystal structure refinement	
Absorption correction, T_{min}, T_{max}	0.435, 1.000	0.645, 1.000
Number of measured, independent and observed [$I > 3\sigma(I)$] reflections	541, 539, 391	678, 416, 320
R_{int}	0.004	0.013
$(\sin\theta/\lambda)_{max}$ ($Å^{-1}$)	1.029	0.847
$R[F^2 > 2\sigma(F^2)]$, $wR(F^2)$, S	0.055, 0.069, 3.60	0.072, 0.195, 1.13
Number of reflections	539	416
Number of parameters	63	63
$\Delta\rho_{max}$, $\Delta\rho_{min}$ ($e{\cdot}Å^{-3}$)	1.10, -1.22	1.79 / -1.7
The shortest Fe-Fe distances in the prismatic and octahedral chains		
Short Fe1_1–Fe1_2, Å	2.6099	2.5684
Long Fe1_1–Fe1_2, Å	2.8185	2.7604
Short Fe2_1–Fe2_1, Å	2.5389	2.5031
Long Fe2_1–Fe2_1, Å	2.8902	2.8265
Short Fe3_1–Fe3_2, Å	2.5628	2.5237
Long Fe3_1–Fe3_2, Å	2.8663	2.8057

Atomic coordinates

Atom	Site	x/a	y/b	z/c	x/a	y/b	z/c
Fe1_1	2e	0.0237(4)	0.25	-0.0042(9)	0.0314(6)	0.25	-0.0014(4)
Fe1_2	2e	0.0289(5)	0.25	-0.4837(9)	0.0346(6)	0.25	-0.4826(4)
Fe2_1	4f	-0.0045(4)	0.4992(7)	0.7327(2)	-0.0050(3)	0.4992(1)	0.7336(3)
Fe3_1	4f	-0.4725(3)	0.3839(5)	0.3683(6)	-0.4676(4)	0.3842(2)	0.3701(3)
Fe3_2	4f	-0.4635(3)	0.3857(5)	-0.1015(6)	-0.4585(4)	0.3847(2)	-0.1012(3)
O1	4f	-0.7275(13)	0.451(3)	0.061(2)	-0.724(2)	0.4563(7)	0.0660(12)
O2	4f	-0.7064(15)	0.449(3)	0.573(3)	-0.705(2)	0.4533(8)	0.5775(12)
O3	2e	0.3262(18)	0.25	0.319(3)	0.332(3)	0.25	0.3309(16)
O4	4f	-0.1578(14)	0.3547(19)	0.206(3)	-0.155(2)	0.3530(8)	0.2150(12)
O5	4f	-0.1775(14)	0.3534(18)	-0.303(2)	-0.173(2)	0.3525(8)	-0.2959(11)
O6	2e	0.3390(17)	0/25	-0.179(3)	0.342(3)	0.25	-0.1654(17)

* This study was carried out at the P02.2 beamline at DESY (Hamburg) using a wavelength of λ = 0.2887Å

** This study was carried out at the ID09A beamline at ESRF (Grenoble) with a radiation wavelength of 0.41513 Å.

Supplementary Table 5. Hyperfine parameters from fits to the Mössbauer spectra of Fe_4O_5.

Temperature (K)	Quadrupole shift (mm/s)		
	2.5 GPa	5.5 GPa	8 GPa
295	0.21110		
250	0.22575		
200	0.22965	0.23035	0.18680
150	0.20615	0.16775	0.14165
100	0.02840	0.05500	0.01300
70	0.00635	-0.02300	-0.03365
50			-0.07110
20	-0.06525	-0.08455	-0.08940
