# Supplementary material for: Pressure tuning of charge ordering in iron oxide
Source: Nat Commun. 2018 Oct 8;9:4142. doi: 10.1038/s41467-018-06457-x (PMC6175922; doi:10.1038/s41467-018-06457-x)
Supplement: Supplementary file 2 — Description of Additional Supplementary Files [file 41467_2018_6457_MOESM2_ESM.pdf]

Description of Additional Supplementary Files

File Name: Supplementary Data 1
Description: Crystallographic Information file for the crystal structure of Fe4O5-III refined at 180 K and 11.7 GPa in C2/m symmetry

File Name: Supplementary Data 2
Description: Crystallographic Information file for the crystal structure of Fe4O5-III refined at 180 K and 11.7 GPa in C2221 symmetry

File Name: Supplementary Data 3
Description: Crystallographic Information file for the crystal structure of Fe4O5-IV refined after laser heating at 48 GPa

File Name: Supplementary Data 4
Description: Crystallographic Information file for the crystal structure of Fe4O5-IV refined at 120 K and 25 GPa

File Name: Supplementary Data 5
Description: Crystallographic Information file for the original crystal structure of Fe4O5–I refined at normal conditions
